# Nonoperative Management of Boerhaave Syndrome: A Case Study

**DOI:** 10.7759/cureus.72573

**Published:** 2024-10-28

**Authors:** Maria Konstantina Tzioti, Alexandra Marinou, Theodoros Sidiropoulos, Anastasia Karachaliou, Nikolaos Danias

**Affiliations:** 1 School of Medicine, National and Kapodistrian University of Athens, Athens, GRC; 2 4th Department of Surgery, "Attikon" University Hospital, National and Kapodistrian University of Athens, Athens, GRC; 3 2nd Department of Radiology, "Attikon" University Hospital, National and Kapodistrian University of Athens, Athens, GRC

**Keywords:** boerhaave syndrome, esophageal rupture after vomiting, esophageal rupture treatment, nonoperative management of rupture, spontaneous esophageal rupture

## Abstract

Spontaneous esophageal rupture, also known as Boerhaave syndrome, represents an unusual yet clinically significant condition characterized by the rupture of the esophageal wall due to a sudden increase in intraluminal pressure, typically induced by vomiting, concomitant with negative intrathoracic pressure dynamics. This condition poses a challenging clinical entity, presenting high mortality rates, especially when treatment is delayed. Surgical intervention is frequently employed as the primary management strategy, while non-surgical approaches, including stent placement and endoluminal vacuum therapy, are less commonly utilized. This study aims to provide insights into the efficacy of non-operative management strategies by examining a clinical case. A 20-year-old male patient presented with fever, epigastric pain, excessive vomiting, and diarrhea over the past four days. A week prior, he had been examined at a private clinic, diagnosed with acute appendicitis, and managed conservatively with oral antibiotics. On the current admission, a contrast-enhanced thoracoabdominal CT scan revealed pneumomediastinum without fluid collection and portal vein thrombosis evidently due to acute appendicitis. Given the patient’s favorable clinical status and the absence of collections in the mediastinum, a nonoperative approach was decided upon and was eventually successful. This case study concerns the sensible application of conservative modalities in selected patients with esophageal rupture.

## Introduction

First documented in 1723, Boerhaave syndrome was named after Herman Boerhaave, the physician who described the case of a Dutch Grand Admiral, Baron Jan van Wassenaer, who experienced vigorous vomiting following a heavy meal [1]. Three centuries have passed since its first description, yet the rupture of the esophagus remains a critical issue. Boerhaave syndrome is a spontaneous perforation of the esophagus that results from a sudden increase in intraesophageal pressure combined with negative intrathoracic pressure. The most common factors causing this phenomenon include strenuous exertion, vomiting, childbirth, seizures, prolonged coughing, laughing, and weightlifting [2].

Esophageal perforations are a rare occurrence, observed globally among various racial groups, with an estimated annual incidence of 3.1 per 1,000,000 individuals [3]. Notably, this condition exhibits a marked predisposition for men, with male-to-female ratios ranging from 2:1 to 5:1, and a significant proportion of patients having a history of substantial alcohol consumption. Even though the highest risk is observed in men in their sixth and seventh decades, Boerhaave syndrome can also occur in neonates and individuals over 90 years old. Children from 1 to 17 years old are the least affected group. Approximately 15% are classified as spontaneous and typically manifest in patients with intact underlying esophageal anatomy. Nevertheless, a subgroup of Boerhaave syndrome cases presents in patients with underlying conditions such as eosinophilic esophagitis, medication-induced esophagitis, Barrett's esophagus, or infectious ulcers [4].

Esophageal perforations typically involve the left posterior wall of the peripheral intrathoracic esophagus and extend for a few centimeters. However, ruptures may occur in the cervical or intra-abdominal esophagus. The rupture of the thoracic esophagus results in contamination of the mediastinal cavity with gastric contents, leading to chemical mediastinitis with mediastinal emphysema and inflammation, followed by bacterial infection and necrosis of the mediastinum. Perforation above the diaphragm, due to mediastinal inflammation or from the initial perforation, leads to immediate contamination of the subdiaphragmatic cavity and culminates in subdiaphragmatic collection. Left untreated, the patient progresses to sepsis and organ failure, justifying that reported mortality rates approach 60% even with immediate intervention, and rise to 100% if treatment is delayed. This high mortality is primarily attributed to severe local and systemic sepsis stemming from the leakage of esophageal contents [4]. Despite estimating nearly 100% mortality rates in cases without interventional approaches [4], we managed our patient non-operatively, implementing antibiotic therapy and nutritional support, and discharged him after a 12-day stay.

A detailed study of the medical records of a 20-year-old male admitted to the 4th Department of Surgery of Attikon University Hospital with Boerhaave syndrome was performed after obtaining written informed consent.

## Case presentation

A 20-year-old male presented to the ED reporting vomiting for five days, accompanied by persisting fever, with temperatures averaging 38.5°C (101.30°F) and peaking at 40.7°C (105.26°F) earlier that day. Additionally, the patient reported migratory pain, initially periumbilical and later predominantly localized in the hypogastrium. The previous week, he had been examined at a private clinic, complaining of lower abdominal discomfort, mild febrile episodes, and vomiting. A subsequent diagnosis of acute appendicitis was then established based on clinical and imaging findings from a contrast-enhanced CT, and the patient was treated conservatively with oral antibiotic therapy.

Regarding the patient's medical history, he reported a suspected diagnosis of Hashimoto's thyroiditis, which remained unconfirmed. Family medical history did not reveal any notable conditions, and the patient had ceased smoking four months prior, after approximately two pack-years of tobacco use, with no reported alcohol consumption. The patient self-disclosed the use of iron supplements, suggesting potential treatment for anemia. Additionally, the patient had not undergone any screening tests, and a thorough review of systems (ROS) yielded no further findings.

Upon examination, the patient had normal vital signs, with a blood pressure of 124/71 mmHg and a heart rate of 83 beats per minute. Oxygen saturation in ambient air was 96%, alongside a normal body temperature of 36.6°C (97.88°F). Tenderness upon deep palpation of the right upper quadrant was observed.

Investigations

**Figure 1:**
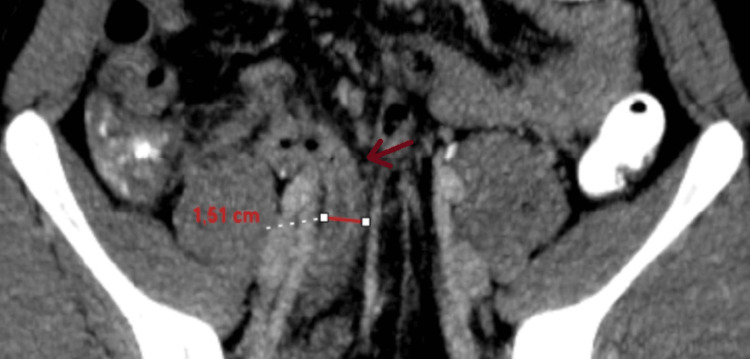
The appendix exhibits features consistent with acute inflammation, including edema, a maximum diameter of 1.5 cm, and thickening of the appendiceal wall.

**Figure 2:**
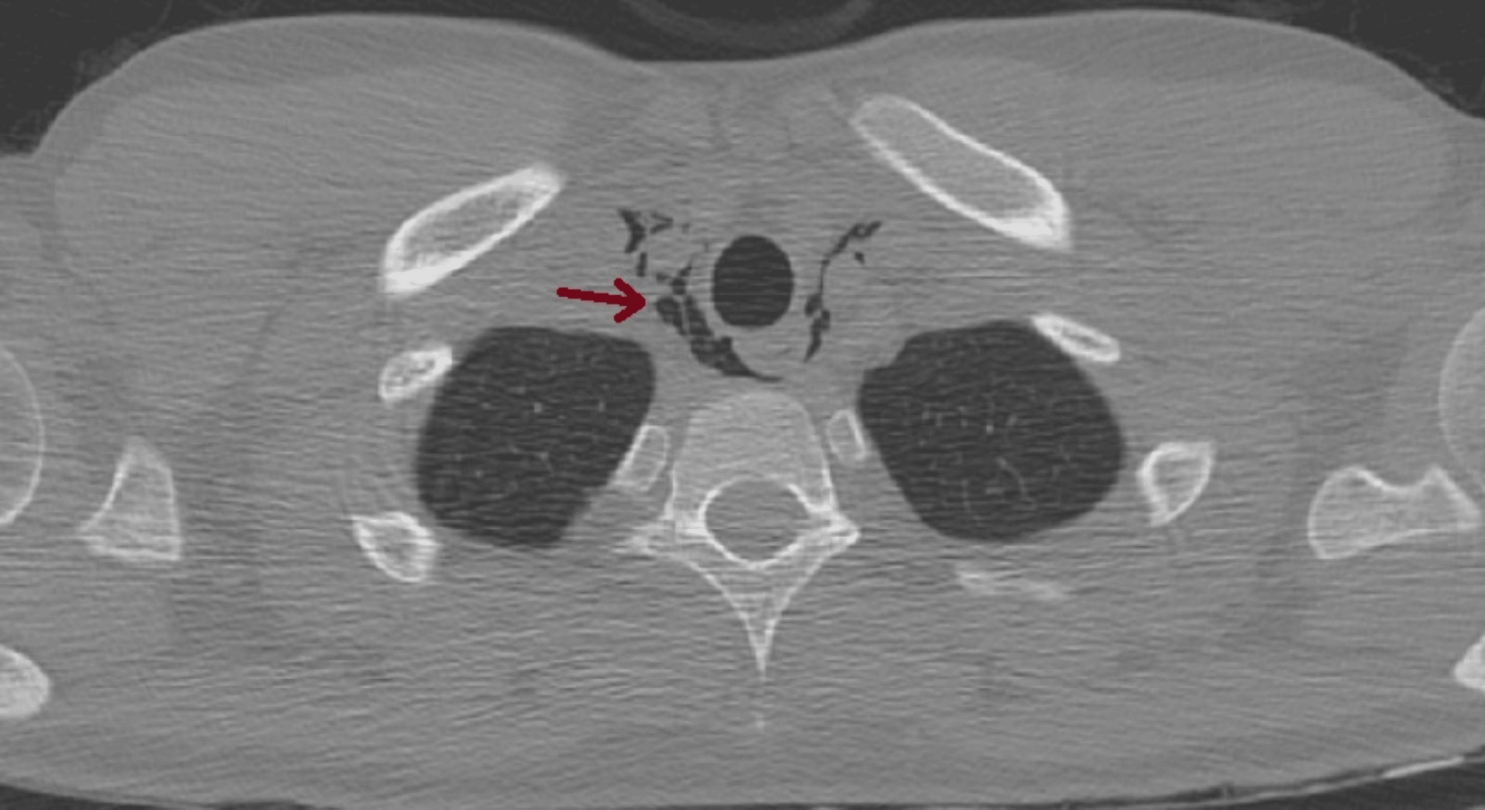
Pneumomediastinum: air bubbles within the mediastinum, adjacent to the trachea, esophagus, and heart.

**Figure 3:**
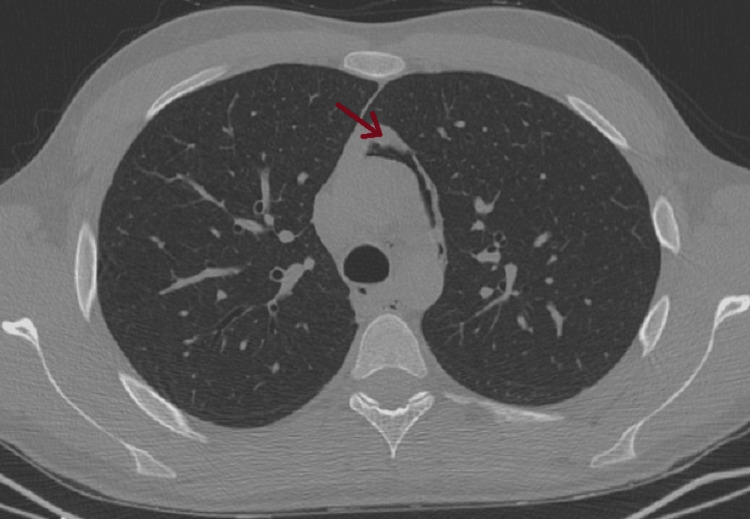
Pneumomediastinum: air bubbles within the mediastinum, adjacent to the trachea, esophagus, and heart.

On admission, laboratory investigations revealed nonspecific abnormalities, including leukocytosis on complete blood count analysis (WBCs 9.18 × 10^9^/L; reference range: 4.00-11.00 K/μL), markedly elevated C-reactive protein (CRP 264 mg/L; reference range: 0.00-6.00 mg/L), elevated fibrinogen (830.5 mg/dL; reference range: 200-400 mg/dL), and D-dimers within normal range (D-dimers 126 ng/dL; reference range: <500 ng/dL). Arterial Blood Gas (ABG) values and other blood test findings were normal. The patient tested negative for SARS-CoV-2. An abdominal ultrasonography (US) showed findings indicating hepatosplenomegaly (liver 18.5 cm and spleen 14.5 cm). To further investigate, a thoracoabdominal CT scan, enhanced with oral and IV contrast, was performed, confirming the diagnosis of appendicitis and revealing the presence of air in the mediastinum, but no evidence of fluid collection (Figures 1-3).

**Figure 4:**
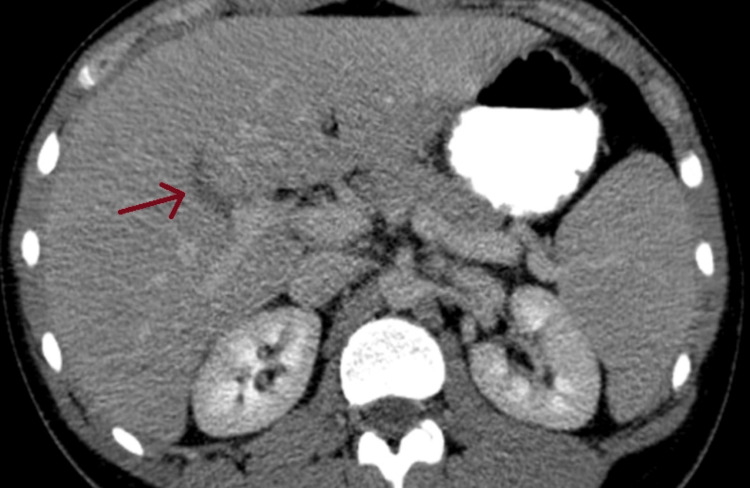
Thrombosis of an intrahepatic branch of the portal vein.

Apart from the pneumomediastinum, there was an incidental finding of portal vein and superior mesenteric thrombosis, evidently due to acute appendicitis (Figure 4).

Treatment

The patient was initially treated conservatively for acute appendicitis, following the latest guidelines and according to the results of the Comparison of Outcomes of Antibiotic Drugs and Appendectomy (CODA) trial [5]. Conservative treatment with empiric antibiotics was continued and escalated due to suspected mediastinitis, including vancomycin, piperacillin/tazobactam, and anidulafungin. The patient remained fasted for eight days, started on peripheral parenteral nutrition (PPN) for two days, and then was transitioned to oral feeding. Additionally, therapeutic doses of enoxaparin (Clexane) were administered to manage thrombophlebitis in the superior mesenteric and right portal veins.

Outcome and follow-up

**Figure 5:**
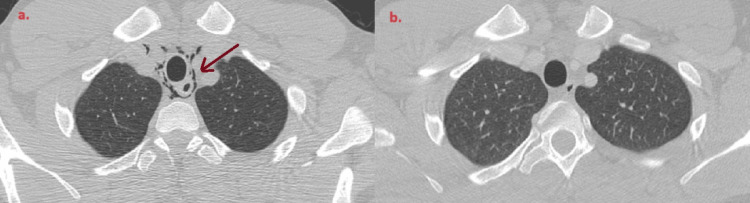
Pneumomediastinum: (a) air bubbles within the mediastinum, adjacent to the trachea and esophagus; (b) Subsidence of pneumomediastinum on follow-up examination three months post-discharge.

**Figure 6:**
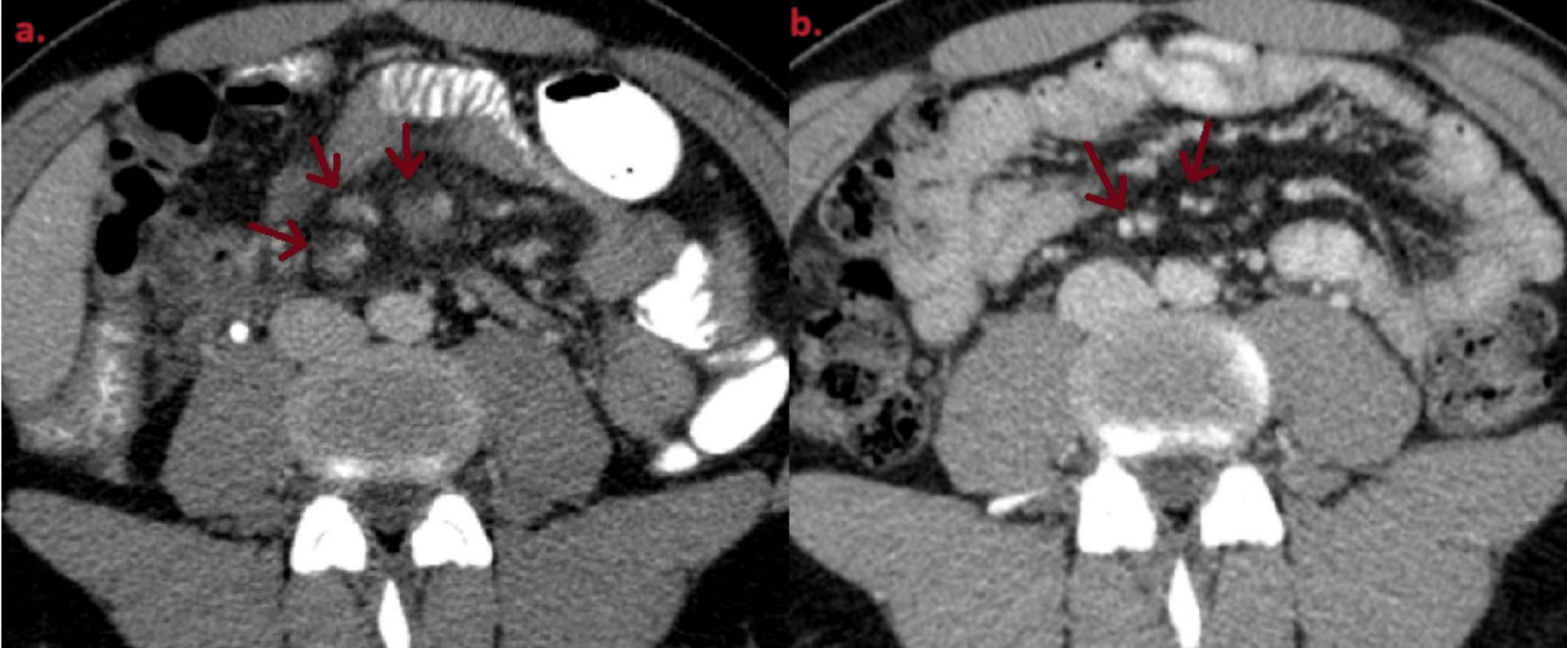
Superior mesenteric vein thrombosis: (a) thrombosis of branches of the superior mesenteric vein; (b) recanalization of branches of the superior mesenteric vein observed after a three-month interval.

The patient successfully completed his antibiotic therapy, leading to the discontinuation of medication, PPN, and IV fluids after a 10-day hospitalization period. Following two days of well-tolerated oral feeding, which indicated successful management of his condition, the patient was discharged from the hospital on the 12th day post-admission. Subsequent testing was negative for thrombophilia. The patient underwent a follow-up CT scan three months post-discharge, after completion of his prophylactic antithrombotic regimen. The re-evaluation showed an improvement in his radiological presentation, indicative of favorable progression (Figures 5-6). As a result, he returned to his everyday activities.

## Discussion

This case is one of the few documented in the international literature wherein a patient with Boerhaave syndrome was managed exclusively with antimicrobial therapy and supportive care, without necessitating surgical or endoscopic intervention.

Maintaining a high level of suspicion is crucial for facilitating early diagnosis, as prompt identification can significantly reduce morbidity and mortality by minimizing mediastinal and pleural contamination, thus preventing sepsis. Differential diagnosis of spontaneous esophageal rupture based on clinical examination findings can be challenging, as the presentation of Boerhaave syndrome with the Mackler triad (comprising vomiting, thoracic pain, and subcutaneous emphysema) occurs in only approximately 50% of cases [6]. The condition can also present with a variety of nonspecific symptoms and signs, leading to misdiagnoses of more common emergency conditions such as aortic dissection, myocardial infarction, pericarditis, myocarditis, pneumonia, pneumothorax, pulmonary embolism, tracheal rupture, perforated peptic ulcer, pancreatitis, Mallory-Weiss syndrome, etc. [4]. In addition, other factors contributing to increased intra-esophageal pressure, such as belching, should also be taken into consideration [7].

CT imaging is the preferred diagnostic modality for Boerhaave syndrome and its associated complications, but other imaging modalities, such as fluoroscopic studies (Esophagram) and endoscopy of the esophagus, substantially contribute to the diagnosis of the condition [7].

Imaging plays a significant role in diagnosing the condition, and it is important to mention some of the imaging findings indicative of Boerhaave syndrome. The first indicator is pneumomediastinum, as seen in a plain chest X-ray or a thoracic CT. Apart from pneumomediastinum, a plain X-ray of a patient suffering from Boerhaave syndrome can show left pleural effusion, left pneumothorax, or gas in the soft tissue of the chest wall and neck. These findings are non-specific, and sometimes the chest X-ray can be normal [8]; thus, CT imaging is required. The diagnosis of Boerhaave syndrome is suggested by observing some of the findings listed in Table 1 [9].

**Table 1:** CT findings indicating Boerhaave syndrome.

CT findings indicating Boerhaave syndrome	
Pneumomediastinum	
Pneumothorax (usually left-sided)	
Pleural effusion (usually left-sided)	
Periaortic gas tracks	
Mediastinal fluid collections	
Esophageal wall thickening	
Gas within soft tissue spaces of the chest wall and neck, around great vessels	
Gas extending into spinal epidural, peritoneal, and retroperitoneal spaces	
Oral contrast extravasation from the esophagus	

A differential diagnosis of this imaging is yet to be made, as some of the above findings can be observed in conditions such as spontaneous pneumomediastinum due to smoking, tobacco use, or drug inhalation, secondary to excessive vomiting (Boerhaave syndrome), excessive cough (asthma exacerbation), increased intrathoracic pressure (forceful sneezing), lung diseases (chronic obstructive pulmonary disease, bronchiectasis, lung cancer), foreign body in the airway, childbirth, iatrogenic causes (endoscopy, intubation), and trauma. Clinical correlation is necessary to establish the diagnosis and proceed to treatment [10].

Our 20-year-old patient did not present with the typical features of spontaneous esophageal rupture. Despite the presence of air and inflammation in the mediastinum, no leakage of contrast material from the esophagus was observed on thoracic computed tomography. Given the patient’s presentation, imaging findings, and course, esophageal rupture was considered the most likely cause of the pneumomediastinum.

While surgical intervention remains the gold standard, limited data support non-operative management. Our initial search for cases similar to ours in databases such as PubMed and Scopus yielded no significant results, indicating that documented cases are scarce in this specific patient population. Much of the existing literature focuses on older adults with risk factors like alcohol consumption, leaving younger, otherwise healthy individuals less represented, as well as individuals diagnosed early without evidence of mediastinal soiling [7]. This case contributes to the ongoing discussion regarding non-surgical treatment options, particularly for younger patients.

Endoscopic stent placement is a valuable alternative that has been demonstrated to be effective and safe for select patients [11]. However, in the present case, this intervention was deemed unnecessary as there was no contrast leak in the mediastinum. Furthermore, as highlighted by Dasari et al., reliance solely on esophageal stent placement for the management of esophageal perforation does not align with established therapeutic objectives for such cases [12]. Notably, other studies have shown that a substantial proportion of patients undergoing esophageal stent placement received jejunostomy tubes (82%), reflecting the complexity of this approach [13]. Considering the patient's young age and overall condition, a high-morbidity approach, such as extensive surgical intervention, or a safer approach, such as endoscopic stent placement, was not preferred over conservative management under vigilant observation.

Additional therapeutic approaches that have recently been incorporated into the management of esophageal perforation include clipping, suturing, and endoscopic vacuum therapy (EndoVac) [14].

Regardless of the type of invasive procedure utilized for managing the condition, it is essential not to overlook the supportive care of such a critically ill patient, which should include antibiotic therapy, nutritional support, and pleural drainage of the leak and other infected cavities [13].

As indicated by the outcome, this approach was successful; the patient improved and was discharged after 12 days. As evidenced by the comparison of CT scans presented in Figure 5, there is a distinct improvement in the patient's radiological presentation. Specifically, the presence of air has notably diminished in the latest images.

## Conclusions

This case study highlights the successful nonoperative management of Boerhaave syndrome in a 20-year-old male patient who initially presented with vomiting and mediastinal air on imaging, suggestive of esophageal rupture. Conservative management was selected, accompanied by careful monitoring. After discharge, follow-up examinations demonstrated notable improvement, affirming the efficacy of the chosen management strategy. Nonoperative management can be a viable and effective approach in select cases of Boerhaave syndrome when the patient is clinically stable, not septic, and there is no contrast extravasation or pleural effusion on imaging. Continued vigilance, interdisciplinary collaboration, and meticulous follow-up are essential for optimizing patient outcomes in such challenging clinical scenarios.
